# Insights into glycogen metabolism in *Lactobacillus acidophilus*: impact on carbohydrate metabolism, stress tolerance and gut retention

**DOI:** 10.1186/s12934-014-0094-3

**Published:** 2014-11-20

**Authors:** Yong Jun Goh, Todd R Klaenhammer

**Affiliations:** Department of Food, Bioprocessing and Nutrition Sciences, North Carolina State University, Raleigh, North Carolina 27695 USA

**Keywords:** Lactobacilli, Glycogen, Probiotic, Raffinose, Gastrointestinal retention

## Abstract

In prokaryotic species equipped with glycogen metabolism machinery, the co-regulation of glycogen biosynthesis and degradation has been associated with the synthesis of energy storage compounds and various crucial physiological functions, including global cellular processes such as carbon and nitrogen metabolism, energy sensing and production, stress response and cell-cell communication. In addition, the glycogen metabolic pathway was proposed to serve as a carbon capacitor that regulates downstream carbon fluxes, and in some microorganisms the ability to synthesize intracellular glycogen has been implicated in host persistence. Among lactobacilli, complete glycogen metabolic pathway genes are present only in select species predominantly associated with mammalian hosts or natural environments. This observation highlights the potential involvement of glycogen biosynthesis in probiotic activities and persistence of intestinal lactobacilli in the human gastrointestinal tract. In this review, we summarize recent findings on (i) the presence and potential ecological distribution of glycogen metabolic pathways among lactobacilli, (ii) influence of carbon substrates and growth phases on glycogen metabolic gene expression and glycogen accumulation in *L. acidophilus*, and (iii) the involvement of glycogen metabolism on growth, sugar utilization and bile tolerance. Our present *in vivo* studies established the significance of glycogen biosynthesis on the competitive retention of *L. acidophilus* in the mouse intestinal tract, demonstrating for the first time that the ability to synthesize intracellular glycogen contributes to gut fitness and retention among probiotic microorganisms.

**Electronic supplementary material:**

The online version of this article (doi:10.1186/s12934-014-0094-3) contains supplementary material, which is available to authorized users.

## Introduction

Glycogen, a soluble multi-branched glucose homopolysaccharide, is a common form of energy storage synthesized by animals and eukaryotic microorganisms. Among prokaryotes, intracellular glycogen has been identified in more than 50 bacterial species including Gram-positive and Gram-negative bacteria as well as archaebacteria (reviewed in [1]). Based on a recent analysis of 1,202 bacterial genomes, intact glycogen metabolic pathways are generally present in species adaptable to more diverse habitats and flexible lifestyles [2]. Structurally analogous to amylopectin, an energy storage component of starch in plants, glycogen is composed of chains of α-1,4-linked glucose units interconnected by comparatively more extensive α-1,6-linked branches. Most bacterial glycogen has an average chain length of ~ 7 to 13 glucose units and an estimated molecular size of ca. 10^7^ to 10^8^ Da [2,3]. Glycogen is considered a flexible and efficient form of energy storage due to its large molecular mass and highly branched structure, and its accumulation has little effect on the internal osmotic pressure of the cells.

**Figure 1:**
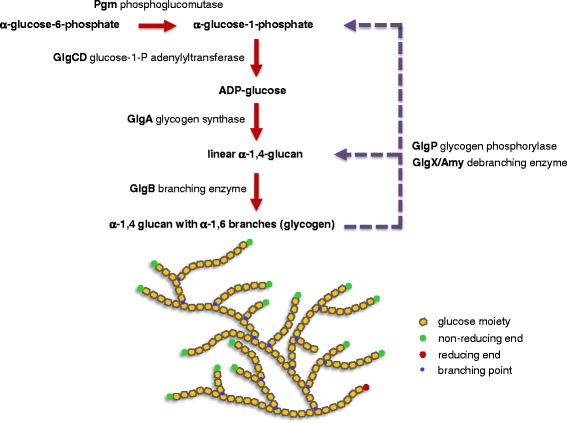
**Classical pathway of glycogen metabolism in prokaryotes.** Pgm converts glucose-6-phosphate into glucose-1-phosphate, which serves as a substrate for ADP-glucose synthesis catalyzed by glucose-1-phosphate adenylyltransferase (GlgCD) encoded by *glgC* and *glgD* genes. Then, GlgA catalyzes the transfer of glucosyl moieties from ADP-glucose to the elongating chain of linear α-1,4-glucan; GlgB subsequently cleaves off a portion of the glucan and attaches it to existing chains via α-1,6 linkages to form the glycogen structure. For glycogen degradation, GlgP sequentially releases glucose moieties from the non-reducing ends to form glucose-1-phosphate and α-1,6-branched dextrins. GlgX or Amy hydrolyzes the α-1,6 branches of the phosphorylase-limit dextrins (typically 3–5 glucosyl residues in length) leading to the release of maltodextrins. Glycogen biosynthetic steps are indicated in red arrows, whereas glycogen degradation pathway is indicated in dashed purple arrows.

Glycogen synthesis in bacteria is generally regarded as a mechanism to prolong survival by supplying energy production during growth-limiting conditions. This so called “energy of maintenance” allows cells to sense and respond to nonsupportive environments, such as starvation and stress [4]. Glycogen may be synthesized during exponential growth or in stationary phase, and its accumulation generally occurs in the presence of excess carbon sources [4]. The classical biosynthetic pathway involves phosphoglucomutase (Pgm), glucose-1-phosphate adenylyltransferase (GlgC or GlgCD), ADP-glucose-specific glycogen synthase (GlgA) and branching enzyme (GlgB) (Figure 1). Phosphoglucomutase converts glucose-6-phosphate into glucose-1-phosphate, which serves as a substrate for ADP-glucose synthesis catalyzed by GlgC or GlgCD. Then, GlgA catalyzes the transfer of glucosyl units from ADP-glucose to the elongating chain of linear α-1,4-glucan. GlgB subsequently cleaves off portions of the glucan and links it to internal glucose molecules in existing chains via α-1,6 glycosidic bonds to form the glycogen structure. The catabolism of glycogen is mediated by glycogen phosphorylase (GlgP) [5] and debranching enzyme (GlgX or Amy) [6], which catalyze the sequential phosphorolysis of α-1,4-glucosyl linkages in the glucan chain from the non-reducing ends and debranching of the limit dextrins generated by GlgP, respectively.

Although the precise function of bacterial glycogen is not well-defined, an increasing number of studies have revealed the involvement of glycogen metabolism in major physiological roles, beyond the synthesis of energy reserve compounds (Table 1). Eydallin *et al*. showed that in *Escherichia coli*, glycogen metabolism is interconnected with global cellular processes including energy production, nutrient transport and metabolism, cell envelope integrity, protein turnover, stress responses and intercellular communication, and is tightly regulated by nutritional and energy status [7,8]. Glycogen synthesis also plays a role in the sporulation of *Bacillus subtilis* [9] and the biosynthesis of trehalose in *Corynebacterium glutamicum* which contributes to osmoprotection and cell wall synthesis [10,11]. In *Mycobacterium smegmatis* and several other microorganisms, the parallel synthesis and degradation of glycogen during early growth phase suggests that the glycogen metabolic pathway functions as a carbon capacitor that regulates downstream carbon and energy fluxes [6,11,12]. The ability to synthesize glycogen has also been associated with the colonization persistence of *Streptococcus mutans* [13], *Mycobacterium tuberculosis* [14] and *E. coli* [15], indicating glycogen synthesis as an important niche factor in the host environments.

**Table 1:** **Functional roles of glycogen metabolic pathway in bacteria**

**Species**	**Roles of glycogen storage/metabolism**	**References**
*Bacillus subtilis*	Sporulation	[9]
*Corynebacterium glutamicum*	Synthesis of trehalose, which is involved in cell wall synthesis and osmoprotection	[10,11,16]
*Escherichia coli*	Intestinal colonization in mice	[15]
*Mycobacterium smegmatis*	Carbon capacitor for glycolysis	[12]
*Mycobacterium tuberculosis*	Capsular glucan synthesis and persistence in mice	[14]
*Propionibacterium freudenreichii*	Long term survival and activity under low temperatures	[17]
*Salmonella enteritidis*	Biofilm formation and virulence	[18]
*Streptococcus mutans*	Persistence in oral cavity and formation of dental caries	[13,19,20]
*Synechococcus elongatus*	Tolerance to salt and oxidative stress	[21]
*Vibrio cholerae*	Environmental persistence and host transmission	[22]

*Lactobacillus acidophilus* was the first probiotic microorganism demonstrated to possess a functional glycogen biosynthetic pathway [23]. This ubiquitous probiotic microbe is widely used in the manufacture of yogurt, fermented dairy products and probiotic supplements [24]. Probiotic attributes of *L. acidophilus* include the alleviation of lactose intolerance and cold and influenza-like symptoms [25,26], the modulation of immune cell functions [27] and the alleviation of abdominal pain via modulation of visceral pain perception [28]. Due to its Generally Regarded As Safe (GRAS) status and the ability to survive transit through the digestive tract, *L. acidophilus* has been considered an ideal vehicle for mucosal-targeted delivery of vaccines and biotherapeutics [29,30]. As with other probiotic microbes, research has revealed *in vivo* mechanisms involved in their survival and interaction with the host to promote biodelivery and fitness in the gut. The presence of intact glycogen metabolic gene clusters in *L. acidophilus* NCFM and certain *Lactobacillus* species typically associated with natural or mammalian host environments led to our speculation that glycogen metabolism potentially contributes to the survival and probiotic functionalities of lactobacilli in the gastrointestinal (GI) tract.

This review highlights our recent findings on the genetics and physiology of glycogen metabolism in *L. acidophilus* [23], including factors (e. g. type and availability of carbon source, growth phase) which affect gene expression and glycogen biosynthesis, and the influence of glycogen metabolism on various probiotic-associated phenotypes of *L. acidophilus*. We also present our most current *in vivo* studies demonstrating that the capability of synthesizing intracellular glycogen contributes to the competitive retention of *L. acidophilus* in the mouse GI tract.

### Glycogen metabolic pathway genes among *Lactobacillus*

**Figure 2:**
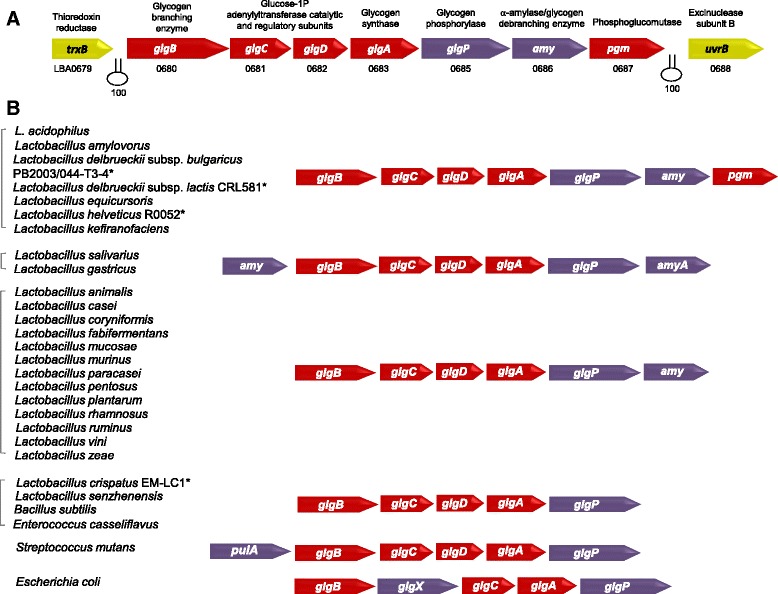
**Genetic organization of glycogen metabolic (** ***glg*** **) operons in** ***Lactobacillus*** **species. (A)** Organization of the glycogen metabolic genes encoded in *L. acidophilus* NCFM. Predicted rho-independent transcriptional terminators are indicated in hairpin loops with overall confidence score (ranges from 0 to 100) [31]. **(B)** Comparative gene mosaic arrangement of the *glg* operons among *Lactobacillus* and other representative microorganisms. All *glg* operons in *Lactobacillus* have the signature *glgBCDAP* core genes. In addition, species closely related to *L. acidophilus* have *amy* and *pgm* genes proceeding the core genes. Strain designations were included for specific *Lactobacillus* strains (indicated with asterisks, *) with intact *glg* operons but belong to species that are generally lacking the pathway based on the NCBI genome database to date. Putative glycogen biosynthesis genes are indicated in red arrows, whereas glycogen degradation genes are indicated in purple arrows. *amyA* or *pulA*, α-amylase/pullulanase/glycogen-debranching enzyme. Figure modified from [23].

The glycogen metabolic pathway in *L. acidophilus* is encoded by a 11.7-kb chromosomal region consisting of *glgBCDAP-amy-pgm* genes (LBA0680 to LBA0687) (Figure 2A) [23]. All seven genes are co-transcribed as a polycistronic mRNA and the gene cluster designated as the *glg* operon. To date, among the genome sequences of *Lactobacillus* species available in the NCBI genome database [http://www.ncbi.nlm.nih.gov/genome/browse/], only ~ 30% of the species possess complete glycogen metabolic gene sets. The *glg* operons identified in these *Lactobacillus* species have a conserved chromosomal mosaic arrangement of the *glg* genes, with the *glgBCDAP* genes representing the core genes in these species (Figure 2B). In terms of sequence homology, the glycogen pathway enzymes of *L. acidophilus* exhibited 41 to 90% identity to the corresponding proteins among the other *Lactobacillus* species, with closest orthologs found in *Lactobacillus amylovorus*, *Lactobacillus helveticus* R0052, *Lactobacillus delbrueckii* subsp. *bulgaricus* PB2003/044-T3-4, *L. delbrueckii* subsp. *lactis* CRL581, *Lactobacillus kefiranofaciens* and *Lactobacillus equicursoris*. Phylogenetic analysis of the conserved glycogen synthase, GlgA, showed a phylogenetic relationship with resemblance to that of the 16S rRNA gene sequences of *Lactobacillus* species [23]. This implied that the presence of this operon among specific lactobacilli was not due to recent horizontal gene acquisition.

The *glg*-encoding *Lactobacillus* species predominantly originate from mammalian hosts or natural environments, such as *Lactobacillus salivarius*, *Lactobacillus ruminus*, *Lactobacillus plantarum*, *Lactobacillus rhamnosus*, *Lactobacillus gastricus*, *Lactobacillus mucosae* and *Lactobacillus murinus*. Despite the general absence or degeneration of the gene cluster among the sequenced strains of *L. helveticus* and *L. delbrueckii*, both of which are commonly associated with domesticated dairy environments, the non-dairy origin strains *L. helveticus* R0052, a commercial probiotic strain isolated from sweet acidophilus milk [32], along with a human vaginal isolate of *L. delbrueckii* subsp. *bulgaricus* (PB2003/044-T3-4) and a cheese isolate of *L. delbrueckii* subsp. *lactis* (CRL581) appeared to harbor complete sets of glycogen metabolic pathway genes. This observation reflected a niche-specific function of glycogen metabolism among lactobacilli. The conservation of the genomic region flanking the *glg* operons in *L. helveticus* R0052 and *L. acidophilus* demonstrated that this operon was lost from the other sequenced strains of *L. helveticus*. In the case of *L. delbrueckii* subsp. *bulgaricus*, small remnants of the *glg* operon were found among the majority of the sequenced strains at the genomic region corresponding to that of the *glg* locus in strain PB2003/044-T3-4. This suggested that only the latter strain preserved an intact *glg* operon as part of a niche-specific gene repertoire equipped for its adaptation to the human vaginal environment.

It is noteworthy that, most *Bifidobacterium* species known as dominant GI commensals and probiotic microorganisms also possess all the essential enzymes for glycogen biosynthesis and catabolism [2]. The glycogen pathway genes in *Bifidobacterium* species, like in *C. glutamicum* [16], another species of Actinobacteria, are not organized as a single operon but instead are dispersed within the genomes [23]. Meanwhile, no glycogen metabolic genes were found in several species that are closely related to *L. acidophilus* and are commonly associated with mammalian host niches, notably *Lactobacillus gasseri*, *Lactobacillus johnsonii* and the majority of the *Lactobacillus crispatus* strains. Nonetheless, the presence of intact glycogen metabolism gene sets in *L. acidophilus* and other *glg*-encoding *Lactobacillus* species, in specific host-associated strains of *L. helveticus* and *L. delbrueckii* subsp. *bulgaricus*, as well as its prevalence in bifidobacteria, indicates a functional and ecological advantage conferred by their ability to synthesize glycogen.

### Glycogen metabolism in *L. acidophilus* is dependent on carbon source and growth phase

The differential expression of the *glg* operon and glycogen accumulation profiles under various carbohydrate conditions demonstrated that glycogen biosynthesis in *L. acidophilus* is highly dependent on the type of sugar substrates present [23]. From a previous microarray-based global transcriptomic study of carbohydrate utilization by *L. acidophilus*, expression of the *glg* operon was upregulated when raffinose or trehalose was provided as a sole carbon source compared to other sugars; whereas glucose appeared to repress the *glg* genes [33]. Subsequently, real time-quantitative PCR (RT-qPCR) experiments and quantitative glycogen assays showed that raffinose was among the sugar substrates examined that induced the highest level of the *glg* operon expression and intracellular glycogen accumulation, followed by the disaccharides trehalose and lactose [23]. Both *glg* expression and glycogen biosynthesis were repressed by glucose. This is consistent with the identification of a catabolite response element (*cre*) upstream of the *glg* operon which is further evidence that glycogen metabolism is subject to catabolite regulation [33]. On the contrary, in microorganisms such as *C. glutamicum*, *S. mutans* and *Salmonella enteritidis*, glycogen accumulation and gene expression were induced in the presence of glucose [16,18,19]. From the perspective of niche adaptation in the small intestinal environment, we theorized that the presence of glucose may signal for nutrient abundance and ideal conditions for population expansion, leading to the prioritization of carbon flow towards glycolysis and other biosynthetic pathways [23]. When glucose is deprived, cells may respond by de-repressing the catabolic machinery for complex carbohydrates along with activating the glycogen metabolic pathway. Under these conditions, glycogen metabolism may generate energy storage reserves and serve as a carbon capacitor in order to regulate downstream metabolic flux, presumably in an energy conservation state to prolong survival.

**Figure 3:**
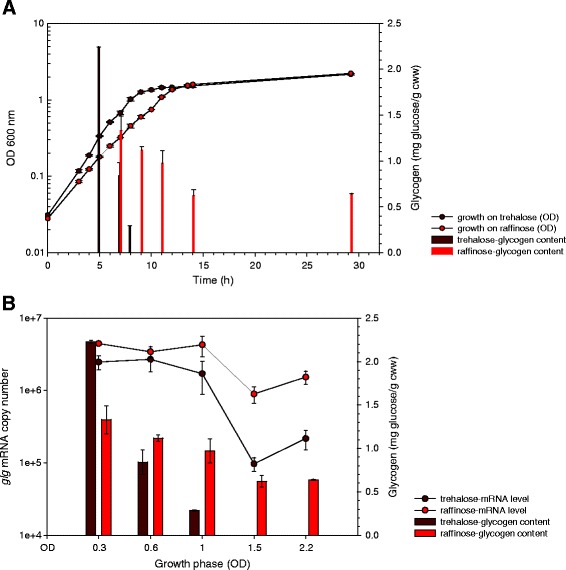
**Intracellular glycogen levels and** ***glg*** **expression during growth on raffinose versus trehalose. (A)** Growth and glycogen accumulation profiles of *L. acidophilus* in semi-defined medium (SDM) [35] containing 2% of raffinose or trehalose as the sole carbon source. The glycogen levels at various growth phases (indicated by OD_600_) from **(A)** were compiled and plotted against the transcript levels of the *glg* operon in **(B)**. The data represent the mean ± standard deviation for two independent biological replicates. Growth phases are represented by OD_600_: 0.3, early log; 0.6, mid-log; 1.0, early stationary; 1.5, stationary, and 2.2, late stationary. Intracellular glycogen content was quantified by hexokinase/glucose-6-phosphate dehydrogenase-based glucose assay and expressed as mg of glucose (released from glycogen by amyloglucosidase) per g of cell wet weight (mg glucose/g cww). Figure adapted from [23].

Temporal glycogen accumulation profiling revealed significant differences during growth on raffinose or trehalose (Figure 3). On both sugars, the highest intracellular glycogen content was observed during early log phase, indicating that *L. acidophilus* was actively synthesizing and accumulating glycogen during the early growth phase. Previous observations in *C. glutamicum* also showed that glycogen accumulated rapidly at early log phase when growing on various sugar substrates [16]. While a dramatic decrease in glycogen level occurred in cells grown on trehalose following early log phase, the raffinose-grown cells maintained at about 50% of their initial glycogen level as the cells entered stationary phase, and remarkably, continued to retain a stable level throughout late stationary phase (Figure 3). Since the glycogen synthesis and degradation genes are co-transcribed, it is likely that the observed glycogen levels were the result of post-transcriptional regulation. It was previously proposed that the balance of parallel synthesis and degradation pathways serves to maintain the glycogen structures and function as a carbon capacitor for sensitive regulation of downstream carbon and energy fluxes [6,11,12,34]. It is plausible that *L. acidophilus* may maintain a higher level of intracellular glycogen as well as *glg* expression to enhance sustainability when growing on a more complex carbon source, a scenario most likely encountered in the gut environment. This presents a novel strategy for utilizing raffinose and potentially other prebiotic oligosaccharides to induce the cells into accumulating and maintaining a stable glycogen pool in order to enhance the viability and residence time of *L. acidophilus* in the GI tract. Meanwhile, it is interesting to note that the transcription of the *glg* operon was elevated in cells cultured in semi-defined medium without a carbon source [23] as well as during late stationary phase (Figure 3B). We speculated this might be partly due to the de-repression of the *glg* operon when fermentable carbon sources are depleted. It is also possible that the *glg* operon was induced during transient starvation conditions, presumably for the catabolism of remaining glycogen storage, or as part of a stress response regulon.

### Functional characterization of the *glg* operon

**Figure 4:**
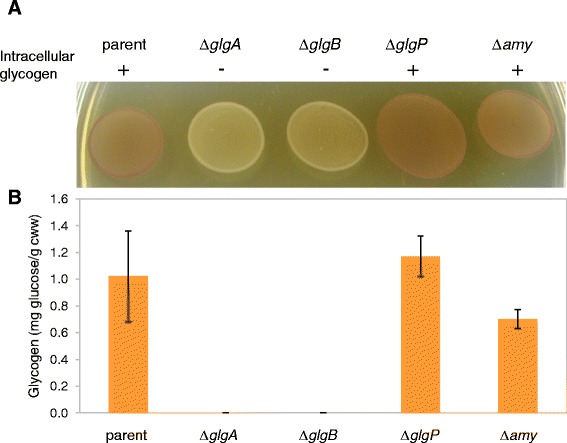
**Role of** ***glgA*** **and** ***glgB*** **in glycogen biosynthesis. (A)** Iodine staining of *L. acidophilus* NCK1909 parent strain and *glg* mutants grown on solid SDM containing 2% trehalose. Both Δ*glgA* and Δ*glgB* mutant cells appeared as yellow/colorless indicative of glycogen-deficient phenotype. Like the parent cells, the Δ*glgP* and Δ*amy* mutants were stained brown, indicating that their ability to synthesize intracellular glycogen was unaffected. **(B)** Quantitative analysis of intracellular glycogen content in mid-log phase cells cultivated in SDM containing 2% trehalose, confirming the absence of intracellular glycogen in both Δ*glgA* and Δ*glgB* cultures. Data shown represent the mean ± standard deviation for two independent biological replicates. Figure adapted from [23].

An in-frame deletion of the *glgA* or *glgB* gene resulted in a glycogen-deficient phenotype in both mutants of *L. acidophilus* (Figure 4). Hence, the inactivation of *glgA* or *glgB* alone abolished the ability of the mutants to synthesize glycogen, confirming that (a) the *glg* operon is functional in *L. acidophilus*; (b) *glgA* and *glgB* encodes for a glycogen synthase and a glycogen-branching enzyme, respectively, and (c) both functional *glgA* and *glgB* are required for the formation of intracellular glycogen. Interestingly, both Δ*glgA* and Δ*glgB* mutants, but not the Δ*glgP* or Δ*amy* mutant, exhibited less robust growth on raffinose compared to the parent strain [23]. These results suggest an interconnection between glycogen synthesis and raffinose metabolism. The growth defect on raffinose might be due to the major influx of glucose-1-phosphate intermediates (resulting from raffinose catabolism) into the glycogen synthesis pathway, leading to the accumulation of ADP-glucose and linear α-glucan chains in the Δ*glgA* and Δ*glgB* mutants, respectively. The inability of the mutants to convert these intermediates into glycogen macromolecules may consequently affect carbon down flow and compromise growth.

Inactivation of the *glgB* or *glgP* gene resulted in slightly slower growth and colony morphotype variation in MRS medium. Both mutants also showed increased sensitivity to bile and simulated small intestinal juice. The bile sensitivity phenotype might be attributed by an overall growth defect which led to decreased efficiency of the bile efflux systems [36], and possibly the functionality of other resistance mechanisms that protect the cells from digestive enzymes (i.e., amylase, protease, lipase) present in the simulated small intestinal juice. Given that the same phenotype was not observed in the other glycogen-deficient mutant, Δ*glgA*, we speculate that in the Δ*glgB* mutant, accumulation of the α-glucan polymers that were unable to form glycogen structures resulted in various physiological perturbations that affected its growth and survival in the small intestinal environment. In *M. tuberculosis*, inactivation of GlgB was lethal which might be due to the intracellular accumulation of poorly soluble linear α-glucan polymers [14]. With respect to the Δ*glgP* mutation, a similar growth defect and morphological alteration was also observed in *M. smegmatis* carrying a mutation in the glycogen-degrading enzyme gene, *glgE* [12]. Based on the authors’ proposed model of glycogen metabolism as a carbon capacitor for glycolysis, the absence of GlgE disrupts glycogen recycling, resulting in glucose molecules sequestered in glycogen, thereby compromising downstream metabolic pathways. Hence, the growth and bile sensitivity phenotypes of the *L. acidophilus* Δ*glgP* mutant emphasize the physiological importance of coordinated glycogen synthesis and degradation as well as the ability of the cells to retrieve a carbon source from glycogen storage during normal growth as well as stress conditions.

### *In vivo* competitive and displacement colonization studies supporting the role of glycogen biosynthesis in gut retention

**Figure 5:**
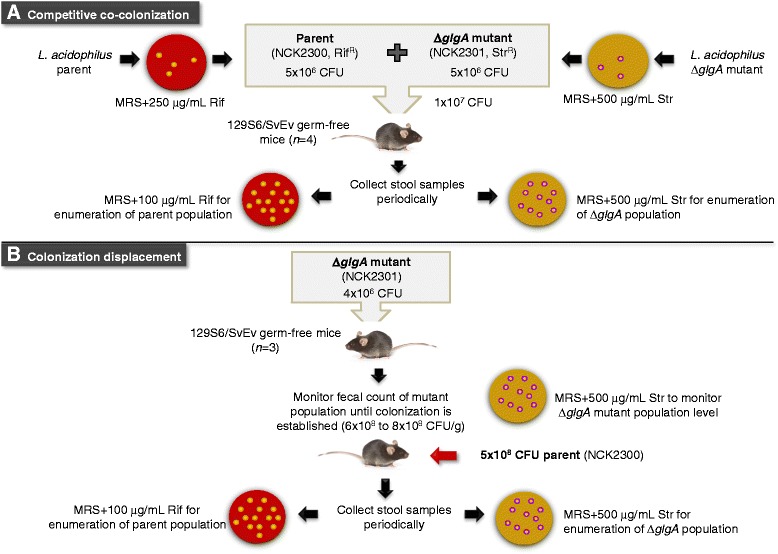
**Competitive co-colonization and colonization displacement experiments** ***in vivo*** **. (A)** Competitive colonization study comparing GI retention of the glycogen-deficient Δ*glgA* mutant with the parent strain. Antibiotic-resistant derivatives of parent and Δ*glgA* mutant were generated for the studies as described in the text. Germ-free 129S6/SvEv mice (2 males, 2 females; 12–26 weeks old) were used in the co-colonization experiment carried out at the North Carolina State University Gnotobiotic Core Facility. Animal use protocols were approved by the Institutional Animal Care and Use Committee of North Carolina State University. Mice were maintained in cages in germ-free flexible film isolators housed in a room with cycles of 12 h of light and darkness, and were provided access to a standard diet (Prolab RMH 3500, LabDiet, St. Louis, MI) and water *ad libitum*. The mice were verified germ-free by culturing of fecal samples aerobically and anaerobically on plate count agar and MRS agar prior to experiments. On Day 0, both parent and Δ*glgA* mutant cells grown overnight in MRS broth were harvested, washed once with phosphate-buffered saline (PBS, pH 7.4; Life Technologies) and resuspended in fresh PBS. The cultures were each titered in PBS and combined in 1:1 ratio to obtain 1 x 10^7^ cells in total per 200 μL of gavage volume. Following gavage (200 μL/mouse), fecal samples were collected periodically, weighed, homogenized in PBS, diluted and plated onto antibiotic selective media for enumeration of the parent and mutant populations. **(B)** Germ-free mice (3 males, 27 weeks old) were first gavaged with the *glgA* mutant. A second gavage with the parent strain was subsequently performed when the *glgA* mutant population reached 6 x 10^8^ – 8 x 10^8^ CFU/g of fecal samples (indicated by a red arrow). Preparation of bacterial cells for gavage and sampling of fecal samples were carried out as described above.

In enteric bacteria such as *E. coli*, glycogen storage plays a critical role in the colonization of the mouse GI tract as evidenced by the significant colonization defect of mutants that are unable to synthesize or degrade internal glycogen [15]. In order to establish whether the ability to synthesize intracellular glycogen contributes to the retention of *L. acidophilus* in the GI environment, we conducted two *in vivo* studies using a 129S6/SvEv germ-free mouse model. The first experiment involved a competitive colonization strategy whereby the germ-free mice were co-colonized with both the Δ*glgA* glycogen-deficient mutant and the parent strain in equal ratios. The GI retention of both strains was monitored by plating fecal samples during a period of 4 weeks (Figure 5). In the follow-up experiment, germ-free mice were first mono-colonized with the Δ*glgA* mutant, followed by subsequent introduction of the parent strain into the same mice to determine whether the ability to generate glycogen reserves would enable the parent population to overturn the dynamic of the established mutant population in the gut.

Prior to the mouse experiment, in order to enable differential enumeration and comparison of the bacterial populations representing the parent (NCK1909) [37] and the Δ*glgA* (NCK2180) [23] strains in the fecal samples, the parent and mutant strains were marked with spontaneous resistance to different antibiotics. Both antibiotics, rifampicin (Rif) and streptomycin (Str), were selected for use due to their low minimal inhibitory concentrations [38] and relatively low spontaneous resistance frequency exhibited by *L. acidophilus*. Both antibiotic resistance phenotypes do not provide cross-protection against each other. One Rif-resistant (Rif^R^) parent and one Str-resistant (Str^R^) Δ*glgA* mutant colonies were isolated from exposure to the respective antibiotics and designated as NCK2300 (parent) and NCK2301 (Δ*glgA* mutant), respectively (Figure 5A and Additional file 1). Both antibiotic-resistant derivatives exhibited undistinguishable growth phenotypes compared to their native counterparts, and the antibiotic resistance phenotypes were stable throughout 30 consecutive passages in the absence of antibiotics, confirming that the spontaneous antibiotic resistant phenotype was stable and did not negatively impact growth (Additional file 1). Since the spontaneous antibiotic resistance phenotype, once established, is inherent in the selected strains, this strategy eliminates the need of external antibiotic agents for maintenance of resistance phenotypes within the bacterial populations in an *in vivo* system. It also allows for the co-investigation of two or more bacterial strains competitively, *in vivo*.

#### Competitive co-colonization of glycogen-deficient ΔglgA mutant and parent strains

**Figure 6:**
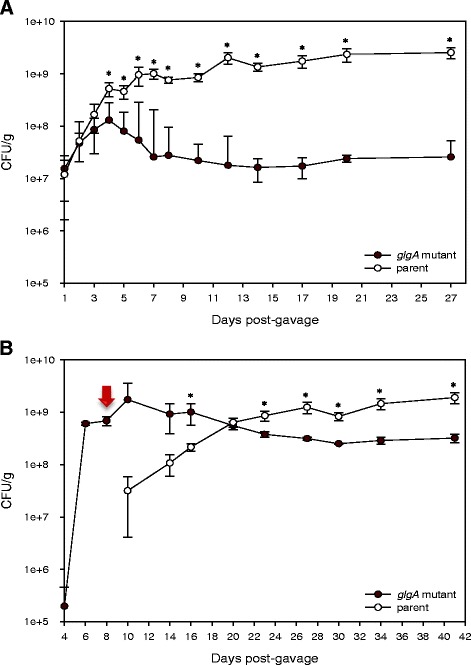
**Co-colonization and colonization displacement studies demonstrating the role of glycogen biosynthesis on competitive gut retention. (A)** Co-colonization of both the glycogen-deficient Δ*glgA* mutant and the parent strain in germ-free 129S6/SvEv mice resulted in an overall 2 log reduction of the Δ*glgA* mutant population. Data shown represent the median cell counts values and mean cell counts ± standard deviation from all four mice. **(B)** Addition of the parent strain to gnotobiotic mice previously mono-colonized with the Δ*glgA* mutant resulted in a population shift with gradual displacement of the mutant population by the parent. Red arrow indicates the timepoint at which the parent strain was administered in a single gavage dose of 5 x 10^8^ CFU. Data shown represent the average cell counts values and mean cell counts ± standard deviation from all three mice. An asterisk (*) indicates a statistically significant difference between the mutant and parent populations (*p*-value < 0.05). Both *in vivo* studies established that a functional glycogen biosynthetic pathway contributes to the competitive advantage and retention of *L. acidophilus* in the GI tract.

On the initial day (Day 0) of the mouse experiment, both parent (NCK2300) and Δ*glgA* mutant (NCK2301) derivatives were combined in 1:1 ratio of cell concentrations and delivered to the germ-free mice (*n* = 4) by intragastric gavage (1 × 10^7^ CFU total in 200 μl) (Figure 5A). Following administration, fecal samples were collected periodically to differentially enumerate the parent and mutant populations on antibiotic selective media. The results in Figure 6A showed an initial population decline of the Δ*glgA* mutant on Day 4 post-gavage. The population level of the mutant continued to decline and was maintained at a 2 log reduction compared to the parent population. We suspect that the lack of a further decline of the mutant population was likely due to the coprophagic nature of rodents. This study demonstrated that the ability of *L. acidophilus* to synthesize intracellular glycogen storage provides a competitive advantage in the mouse GI tract.

#### Colonization displacement of ΔglgA mutant by the parent strain

Germ-free mice (*n* = 3) were initially mono-colonized with the *glgA* mutant by intragastric gavage at a dose of ca. 4 × 10^6^ CFU (Figure 5B). In the absence of competition, the mutant was able to establish initial population levels in the gut (> 6 × 10^8^ CFU/g of fecal samples; Figure 6B) that are at least 5 times higher than its population levels during previous co-colonization studies (highest population count ~ 1 × 10^8^ CFU/g; Figure 6A). The parent strain (ca. 5 × 10^8^ CFU) was subsequently administered to the mice by gavage when the *glgA* mutant population reached the level of 6 × 10^8^ – 8 × 10^8^ CFU/g. The population dynamic of the mutant and parent were monitored as described previously by differential plating of fecal samples on antibiotic selective media. Our data showed that the introduction of the parent strain resulted in a population shift whereby the decline in the mutant abundance coincides with the continual increase of the parent population level (Figure 6B). The observed population displacement of the Δ*glgA* mutant by the parent strain further substantiates the role of glycogen metabolism on the competitive fitness of *L. acidophilus* in the host environment. Moreover, the ability of the Δ*glgA* mutant to establish initial colonization in a germ-free system provided evidence that the decline of the mutant population in the initial competitive colonization study was not due to colonization defect *in vivo*, but rather the lost of competitiveness against the parent population that possesses glycogen biosynthetic capability.

## Conclusions

**Figure 7:**
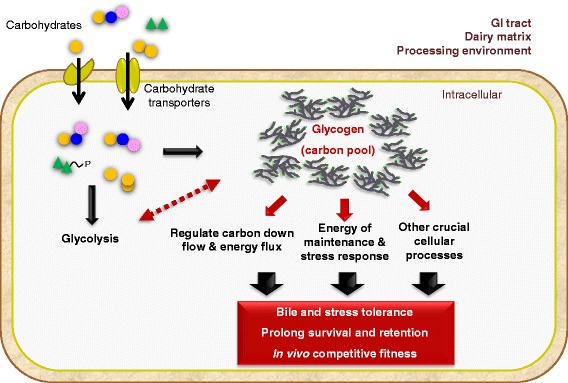
**Proposed mechanisms and functions of glycogen metabolic pathway in** ***L. acidophilus*** **.** Based on proposed central functions of glycogen metabolism in other microorganisms [6,11,12] and findings from this study, carbohydrate substrates imported by the cells may be either metabolized through the glycolytic pathway, or shunted to glycogen biosynthetic pathway when a carbon source is not immediately required for glycolysis. The carbon pool may serve as an energy storage reserve and as a carbon capacitor that senses and modulates downstream carbon flow to maintain efficient carbon utilization and energetic homeostasis of the cells. The roles of glycogen metabolism in central carbon metabolism influence various physiological functions and consequently the retention and probiotic attributes of *L. acidophilus*. Our fundamental understanding of this pathway will inspire strategies to improve the stability and functionalities of probiotic and beneficial commensal microorganisms in the host.

These studies have provided crucial insights into the broad biological functions and probiotic attributes in *Lactobacillus* that rely on functional glycogen biosynthetic and catabolic pathways. Genetic dissection of the pathway in *L. acidophilus* has thus far suggested that glycogen metabolism plays multifaceted roles including normal growth maintenance, the utilization of certain complex sugars (e.g. raffinose), bile tolerance and competitive fitness in the gut. Although the underlying mechanisms on how glycogen metabolism operates globally remains to be established, it is likely that the *in vitro* phenotypes translated to the observed competitiveness of *L. acidophilus*, *in vivo*. Koch [39] previously proposed that bacteria in nature generally exist between the states of feast or famine whereby nutrients are rarely in constant supply. This is supported by the fact that glycogen storage is ubiquitous among enteric bacteria, possibly due to the necessity to support rapid growth in the intestinal environment where there is intense competition for nutrients [15]. In view of this, the ability of *L. acidophilus* to synthesize and store energy in the form of glycogen, either prior to (e.g. during commercial processing or product storage) or during its transit through the host, potentially confers advantages to its retention and probiotic activities in the GI tract. We further postulate that the concerted glycogen anabolism and catabolism in *L. acidophilus* serves to regulate central carbon flow and operate based on nutrient status to conserve energy and resources in the competitive intestinal environment (Figure 7). Overall, we foresee that this work will serve as a foundation to expand our understanding on the roles of glycogen metabolism in other probiotic and commensal species of *Lactobacillus* and *Bifidobacterium*, and to apply the knowledge for improvement of biodelivery and gut persistence of these microorganisms within the host.

## Additional file

Additional file 1:
**Methods on generation of spontaneous antibiotic-resistant derivatives of** ***L. acidopohilus*** **parent and** Δ***glgA*** **glycogen-deficient mutant for** ***in vivo*** **studies, and Figure depicting growth and stability assessment of the antibiotic-resistant phenotypes of NCK2300 (parent) and NCK2301 (Δ** ***glgA*** **mutant).**

## Abbreviations

GI: Gastrointestinal
GlgA: Glycogen synthase
GlgB: Glycogen-branching enzyme
GlgCD: Glucose-1-phosphate adenylyltransferase
GlgP: Glycogen phosphorylase
GlgX: Glycogen-debranching enzyme
Pgm: Phosphoglucomutase
Amy: Putative α-amylase/debranching enzyme
Rif: Rifampicin
Str: Streptomycin
CFU: Colony forming unit
