# Supplementary material for: Insights into glycogen metabolism in Lactobacillus acidophilus: impact on carbohydrate metabolism, stress tolerance and gut retention
Source: Microb Cell Fact. 2014 Nov 20;13:94. doi: 10.1186/s12934-014-0094-3 (PMC4243779; doi:10.1186/s12934-014-0094-3)
Supplement: Additional file 1: — Methods on generation of spontaneous antibiotic-resistant derivatives of L. acidopohilus parent and ΔglgA glycogen-deficient mutant for in vivo studies, and Figure depicting growth and stability assessment of the antibiotic-resistant phenotypes of NCK2300 (parent) and NCK2301 (Δ glgA mutant). [file 12934_2014_94_MOESM1_ESM.pdf]

ADDITIONAL FILES

Additional file 1.pdf

Additional file 1. Methods on generation of spontaneous antibiotic-resistant derivatives of *L. acidopohilus* parent and Δ*glgA* glycogen-deficient mutant for *in vivo* studies.

Additional file 1 Figure. Growth and stability assessment of the antibiotic-resistant phenotypes of NCK2300 (parent) and NCK2301 (Δ*glgA* mutant).

Additional file 1 Methods on generation of spontaneous antibiotic-resistant derivatives of *L. acidopohilus* parent and Δ*glgA* glycogen-deficient mutant for *in vivo* studies.

Methods:

Generation of spontaneous antibiotic-resistant derivatives for *in vivo* studies

For isolation of the rifampicin-resistant (Rif^R) parent (NCK1909) derivative, an overnight culture grown in MRS broth was plated (undiluted) onto MRS agar plates containing 250 µg/mL of rifampicin (Rif) (see Additional file 1 Figure). In order to obtain the streptomycin-resistant (Str^R) Δ*glgA* mutant (NCK2180) derivative, an overnight culture grown in MRS broth was pelleted by centrifugation, resuspended in 1/5 volume of the spent culture supernatant, and the concentrated culture was plated onto MRS agar plates containing 500 µg/mL of streptomycin (Str). All plates were incubated at 37°C for 48-72 hr anaerobically (MRS agar plates containing Rif are light-sensitive and were incubated in the dark). Several antibiotic resistant colonies recovered on each medium were selected and further purified by re-streaking onto MRS plates with the respective antibiotics to obtain pure cultures. One of each Rif^R parent and Str^R Δ*glgA* mutant colony was selected for the growth experiment in MRS broth. Both antibiotic-resistant derivatives exhibited undistinguishable growth phenotype compared to their native counterparts (Additional file 1 Figure), confirming that the spontaneous antibiotic resistance did not have a negative effect on growth.

To further confirm that the Rif and Str resistance phenotypes are stable in the absence of the antibiotics in the growing medium, the Rif^R parent strain (NCK2300) and Str^R Δ*glgA* mutant (NCK2301) were subcultured continuously in MRS broth without antibiotic supplementation for 30 consecutive passages. Aliquots of cultures at certain passages were plated onto both MRS and MRS with the respective antibiotics for each strain. Additional file 1 Figure demonstrated

that the number of cells recovered on antibiotic selective medium was equivalent to those recovered on MRS medium alone for both strains. These results confirmed the stability of the antibiotic resistance phenotypes of both NCK2300 and NCK2301 and that the resistance markers would be suitable for differential enumeration of both populations in the *in vivo* studies.

Additional file 1 Figure Growth and stability assessment of the antibiotic-resistant phenotypes of NCK2300 (parent) and NCK2301 (Δ*glgA* mutant).

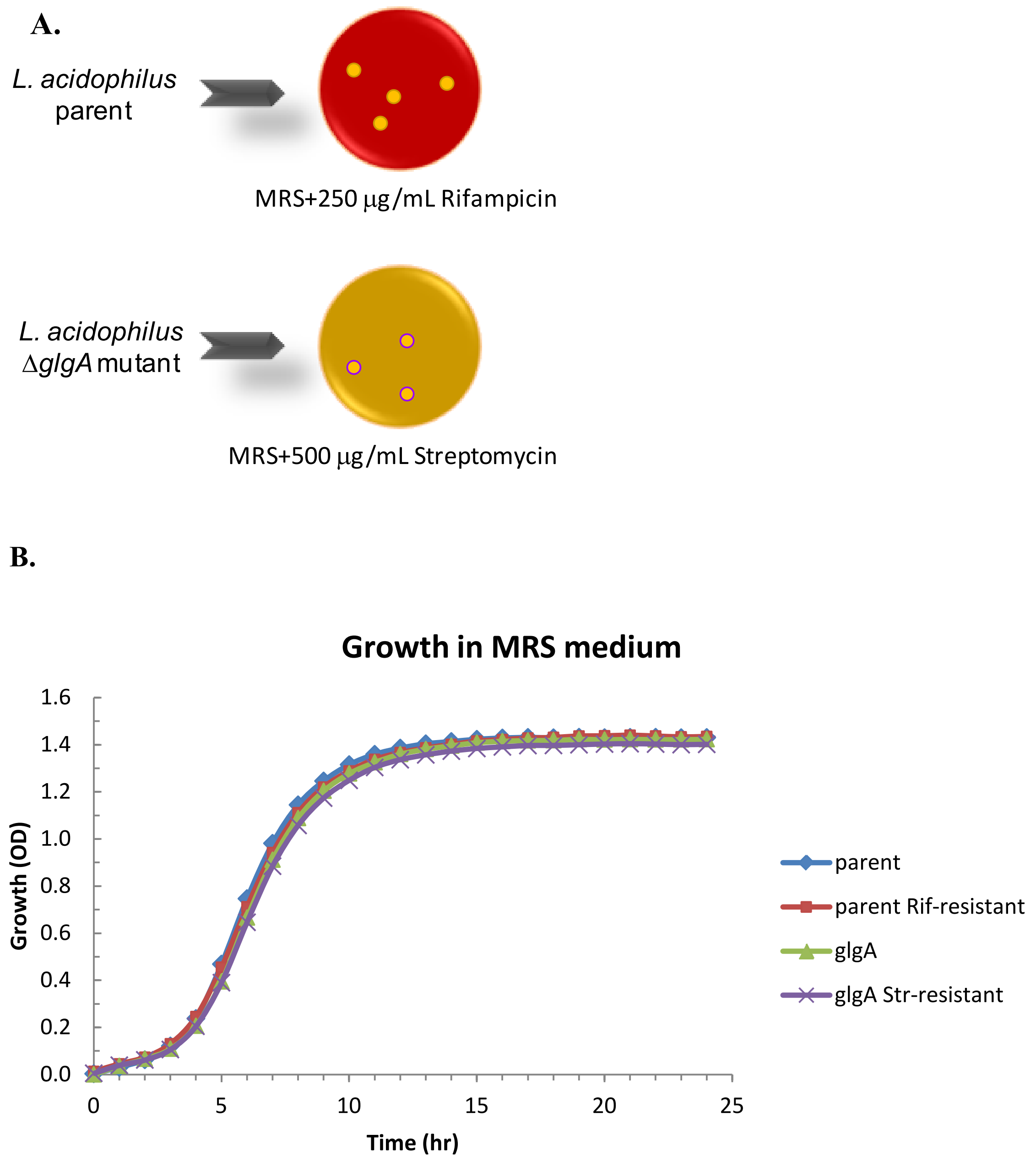

C.

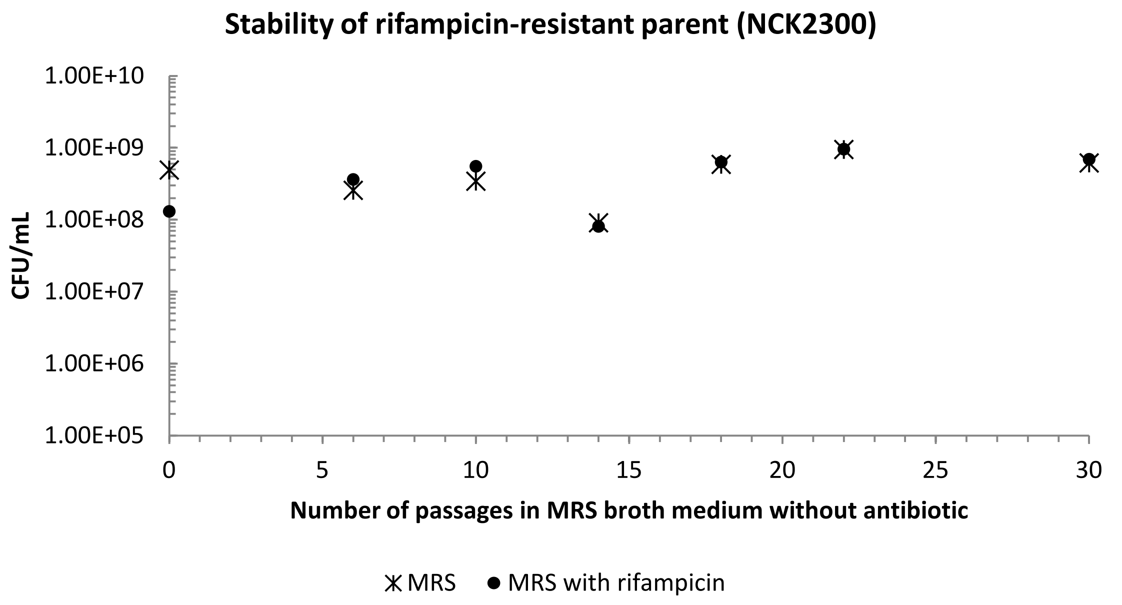
Stability of rifampicin-resistant parent (NCK2300)
CFU/mL
1.00E+10
1.00E+09
1.00E+08
1.00E+07
1.00E+06
1.00E+05
0
5
10
15
20
25
30
Number of passages in MRS broth medium without antibiotic
MRS
MRS with rifampicin

D.

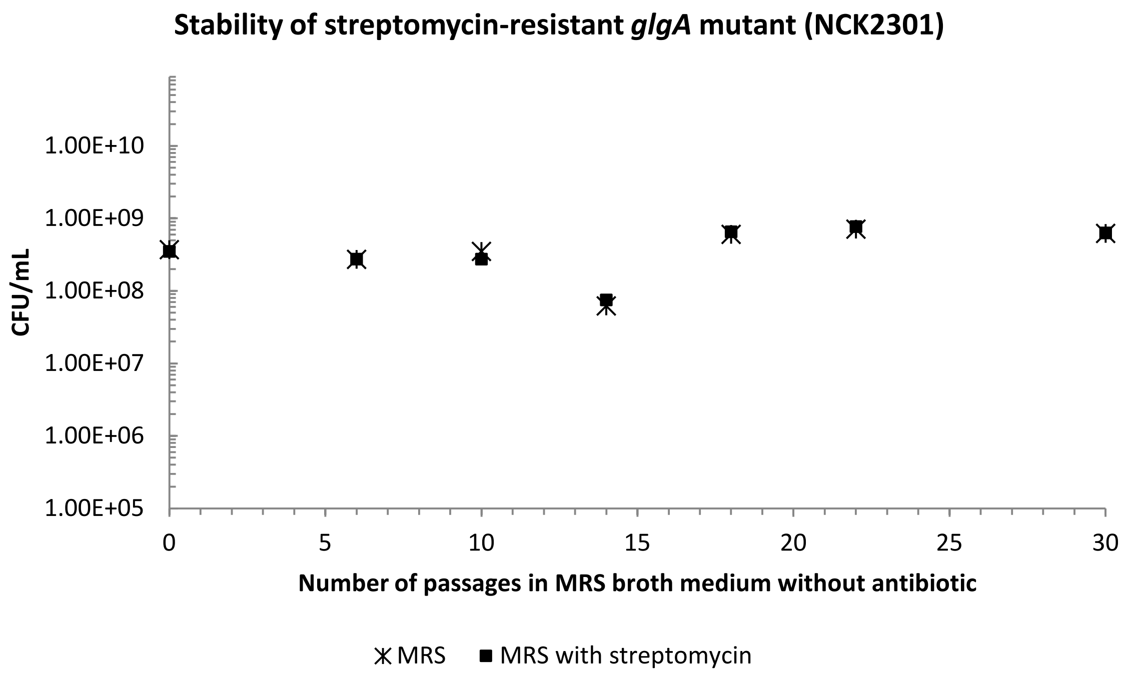
Stability of streptomycin-resistant glgA mutant (NCK2301)
CFU/mL
1.00E+10
1.00E+09
1.00E+08
1.00E+07
1.00E+06
1.00E+05
0
5
10
15
20
25
30
Number of passages in MRS broth medium without antibiotic
MRS
MRS with streptomycin

Additional file 1 Figure (A) Generation of spontaneous rifampicin-resistant (Rif^R) and streptomycin-resistance (Str^R) derivatives of NCK1909 parent and Δ*glgA* mutant, respectively. (B) Both spontaneous antibiotic resistant derivatives showed comparable growth rates with their respective native strains in MRS medium. (C) and (D) Continuous subculturing of both Rif^R parent and Str^R mutant derivatives, designated NCK2300 and NCK2301, respectively, in the absence of antibiotic did not affect the stability of the antibiotic resistance phenotypes, as demonstrated by the comparable cell counts on both MRS medium with or without antibiotic following the passages.
